# Barriers to timely diagnosis and treatment of cutaneous T-cell lymphoma in people with intellectual disabilities

**DOI:** 10.1016/j.jdcr.2025.12.048

**Published:** 2026-02-09

**Authors:** Brigit A. Lapolla, Sara Suhl, Yoni Sacknovitz, Emily R. Gordon, Celine M. Schreidah, Caroline Chen, Ikenna Nebo, Lauren M. Fahmy, Larisa J. Geskin

**Affiliations:** a Department of Dermatology, Columbia University Irving Medical Center, New York, New York; b Columbia University Vagelos College of Physicians and Surgeons, New York, New York

**Keywords:** access to care, clinical trials, cutaneous T-cell lymphoma, delayed diagnosis, health disparities, intellectual disability, mycosis fungoides

## Introduction

People with intellectual disabilities (ID) experience significant healthcare disparities, including lower rates of cancer screening, delayed diagnosis, and reduced access to intensive treatments compared to the general population.^1^, ^2^, ^3^, ^4^ Among cancers, cutaneous T-cell lymphoma (CTCL) illustrates clear challenges associated with these disparities. Mycosis fungoides (MF), the most common form of CTCL, is stratified into progressive clinical stages: patch, plaque, and tumor, with the clinical stage at diagnosis guiding therapeutic strategies and prognosis. Retrospective studies have found between 71% and 92.2% of people are diagnosed with CTCL at stage IA or IB: patch or plaque stage with no nodal involvement.^5^, ^6^, ^7^, ^8^ The time to diagnosis from symptom onset varies, with a median time of 3 years reported. We present a series of people with ID seen at Columbia University Irving Medical Center (CUIMC) in whom CTCL was diagnosed at the tumor stage (stage IIB or higher), requiring systemic first-line therapies. To our knowledge, this is the first case series describing CTCL in people with ID, highlighting the unique diagnostic and therapeutic delays that may lead to profound disparities in this understudied population.

## Case reports

**Fig 1:**
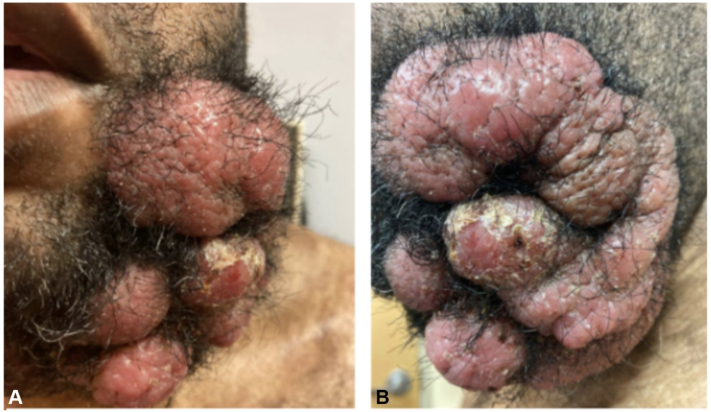
**A** and **B,** Tumor-stage mycosis fungoides. Multilobular, impetiginized tumor measuring 25 × 20 cm on the mandible.

The first patient is a 43-year-old male with ID and Fitzpatrick skin type (FST) V who presented with a 25 × 20 cm ulcerated, impetiginized, multilobular tumor on the mandible and scattered patches, plaques, and small tumors covering 23.5% of the patient’s body surface area (BSA) (Fig 1, *A* and *B*). Six years prior, the patient had developed a pruritic intermittent rash on his extremities that was diagnosed as eczema by an outside dermatologist. Over the course of the next 3 years, this rash progressed in intensity and spread to cover more BSA. The tumor on the left mandible appeared 6 months prior to the patient’s presentation to CUIMC. A diagnosis of tumor-stage granulomatous mycosis fungoides with CD30-negative large-cell transformation was made, 6 years after symptom onset. The patient was treated with pralatrexate 25 mg/m^2^ for 2 months; however, with no decrease in tumor size, treatment with doxorubicin 30 mg/m^2^ was initiated. The patient completed 26 cycles of doxorubicin over the course of 16 months with decrease in tumor size. Imaging for CTCL restaging led to the incidental diagnosis of primary colon cancer, metastatic to the liver, and his family decided to transfer him to a local center for further management.

The second patient is a 64-year-old male with ID and FST V who was diagnosed with mycosis fungoides at the tumor stage, 6 years after symptom onset. At diagnosis, the patient presented with confluent hyperpigmented scaly gray and pink plaques and tumors of varying thickness covering 10.2% BSA, mostly on the buttocks and thighs, with an mSWAT score of 17.8. The patient denied pruritus when asked during the visit, though his caregiver noted he often scratched to the point of bleeding. Excoriations were present on physical exam, confirming the caregiver’s statement. Flow cytometry demonstrated negative blood involvement, and PET-CT revealed numerous low-SUV small bilateral axillary, external iliac, inguinal lymph nodes. After extensive conversations with the patient’s family and caregivers, the decision to pursue narrow-band UVB phototherapy in combination with methotrexate was made. Although bexarotene was seen as the optimal medication for this patient, his residential living center would not have been able to accommodate the optimal fat-restricted diet for this medication. After 2 months on this regimen, the patient experienced additional plaque and tumor development and switched to pralatrexate 30 mg/m^2^. The patient continued to experience disease progression on this pralatrexate and thus began treatment with romidepsin, with some improvement for a few months. After disease progression on romidepsin, treatment options were limited due to his CD30-negativity, prompting consideration of clinical trials. However, initiation of trial therapy was delayed, primarily due to the need for both sponsor and IRB approval for surrogate consent. Further delays occurred while awaiting the patient’s mother, his legal representative, to travel and provide consent in person. The patient was ultimately enrolled in a novel retinoid trial but experienced disease progression soon after the start of treatment. Similar delays were encountered during enrollment into a second trial. Unfortunately, he did not respond to the second treatment either and passed away approximately 2 years after his initial diagnosis.

**Fig 2:**
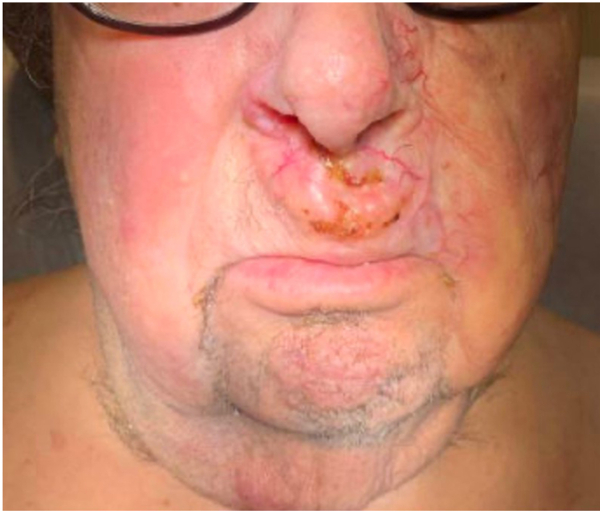
Tumor-stage mycosis fungoides. Impetiginized telangiectatic tumor on upper cutaneous lip.

The third patient is a 80-year-old male with ID and FST I who was diagnosed with folliculotropic tumor-stage mycosis fungoides with large-cell transformation 6 months after experiencing pruritic rashes and a growing tumor on the upper cutaneous lip. The patient was first treated at an outside hospital with methotrexate 20 mg weekly, and mogamulizumab was added after 1 month of methotrexate monotherapy. Upon our initial consultation, 7 months after treatment was initiated, the patient presented with erythematous, telangiectatic tumors on the upper cutaneous lip and left cheek, covering 1.5% TBSA (Fig 2). The patient’s trunk, upper extremities, and lower extremities were covered with scattered erythematous crusted patches and plaques, some with vesiculation and erosions. Treatment options were reviewed during our initial visit; however, the patient’s brother and caregiver expressed that he was unable to bring the patient to CUIMC consistently. Despite multiple outreach attempts, the patient was ultimately lost to follow-up.

## Discussion

Each patient faced many barriers to care throughout his journey with CTCL, often resulting in delayed initiation of treatment. These cases highlight key areas of disparities for people with ID, including delayed diagnosis, reliance on caregivers, and obstacles to clinical trial enrollment, though these barriers are not limited to individuals with ID. These examples offer areas for improvement in diagnostic and therapeutic outcomes for this population.

While delay in CTCL diagnosis is common, especially among individuals with higher FSTs, limited medical care access, and advanced age, this case series also highlights individuals with ID as a vulnerable population.^9^ All 3 patients with ID were diagnosed at the tumor stage, and while we cannot guarantee that each patient’s ID is the cause for this delay in diagnosis, it is clinically suspicious and warrants further attention. Retrospective review of 174 patients with mycosis fungoides at our center revealed that only 4 were diagnosed at tumor stage, and of these 4, 3 (75%) had ID. Given that only about 0.5% to 2% of the general population has ID, it is likely that the delay in CTCL diagnosis among our 3 patients with ID is clinically relevant, though more research is needed.^10^

Diagnostic delay in this population could be explained by differences in symptom communication among individuals with ID, who may express symptoms such as pruritus atypically or not express them at all. For example, one of the patients in this series denied pruritus when asked during his visit, but his caregiver noted that he was constantly scratching to the point of bleeding, while another patient was not able to answer the question at all due to limited verbal abilities. Further, because early lesions often appear in areas typically covered by clothing, they may go unnoticed by caregivers or family members, leading to a delay in medical attention. While this possibility applies to the second patient, whose lesions were mostly on the buttocks and thighs, other patients in our series had facial lesions, suggesting that additional factors beyond clothing-covered sites may also contribute to diagnostic delay Improving clinician and caregiver education about the potential for delayed diagnosis in this population could enhance awareness and clinical suspicion of cutaneous lymphoma. Increased knowledge of how the disease may present, particularly in individuals with ID who may have difficulty communicating symptoms, can prompt more careful evaluation and earlier diagnostic investigations.

Individuals with ID often rely on their family members and caregivers for medical decision making and intervention, which could delay diagnosis and treatment. Logistical barriers can further hinder timely and regular access to medical care. For example, the patient in this case could not continue treatment at the Cutaneous Oncology Program at CUIMC because his brother, who was also his caregiver, could no longer take him to appointments. Such barriers are especially pertinent to treating cutaneous lymphoma, as patients often have to travel long distances to see specialists for advanced disease. While these challenges are difficult to overcome, collaboration with local oncologists and treatment centers could reduce the burden and allow patients to continue receiving specialized cutaneous lymphoma care. Additionally, individuals with ID often rely on their caregivers for communication, which can lead to misunderstandings if their caregivers unintentionally misreports their symptoms. In the case discussed, for instance, the patient denied experiencing pruritus, despite the caregiver reporting frequent scratching and the presence of excoriations on our physical examination. Awareness of these communication limitations is crucial for both clinicians and caregivers, who should remain vigilant for nonverbal signs to supplement verbal reports to ensure accurate assessment and care.

The inclusion of individuals with ID in medical research remains controversial, particularly due to historical abuses such as the Willowbrook Study in the 1960s. When differentiating between therapeutic and non-therapeutic research, the justification for inclusion becomes stronger in the context of therapeutic trials, as these studies often provide direct medical benefits to participants, reducing concerns about exploitation. Still, people with ID are rarely included in therapeutic trials and are sometimes explicitly excluded. A review of 300 high-impact clinical trials found that only 2% explicitly included individuals with ID, while over 90% effectively excluded them.^11^ Although adjustments could have been made to include individuals with ID in at least 70% of these studies, implementing these changes would likely have resulted in delays similar to those experienced by our patient during the trial sponsor and IRB approval processes.^11^ Similarly, a review of NIH-funded clinical trials found that people with ID were directly or indirectly excluded from 75% of trials, with about one-third of the reviewed trials containing language that directly excluded people with ID.^12^ To promote more equitable care, investigators must be willing to go beyond what is administratively convenient, recognizing that additional regulatory measures should not justify the exclusion of patients with ID. Implementing enrollment strategies for individuals with ID, along with surrogate consent procedures at the start of a trial, would likely reduce delays and improve access to therapeutic benefits. However, it is essential that their inclusion adheres to the same rigorous ethical standards applied to all participants: they should not be enrolled in clinical trials ahead of individuals without ID solely based on their disability status, and their inclusion must be grounded in autonomy and informed consent, even if it is a legal representative making those decisions. This issue is particularly relevant in cutaneous lymphoma, where treatment options for advanced-stage disease are limited. Excluding individuals with ID significantly limits their therapeutic options and can accelerate disease progression. More research and discussion are needed to address these disparities for the benefit of both clinical researchers and patients with ID.

These cases highlight the importance of early diagnosis of CTCL and underscore the need for more effective and equitable diagnosis and treatment of cutaneous lymphoma among individuals with ID. They also emphasize the need for discussions about the challenges surrounding clinical trial enrollment for individuals with ID. To our knowledge, there are no reports that discuss cutaneous lymphomas in individuals with ID, and further research is therefore warranted.

## Conflicts of interest

Geskin has served as an investigator for J&J, Mallinckrodt, Kyowa Kirin, Soligenix, Innate, Incyte, Trillium, Merck, BMS, and Stratpharma and on the scientific advisory board for SciTech and Citius.
